# Intrathoracic Breast Transposition: A New Method in the Treatment of Bronchopleural Fistula and Empyema

**DOI:** 10.1097/GOX.0000000000002531

**Published:** 2019-12-30

**Authors:** Louis de Weerd, Petter Cappelen Endresen, Anmar Tabit Numan, Sven Weum

**Affiliations:** From the * Department of Plastic and Reconstructive Surgery, University Hospital of North Norway, Tromsø, Norway; † Medical Imaging Research Group, Department of Clinical Medicine, UiT The Arctic University of Norway, Tromsø, Norway; ‡ Department of Cardiothoracic Surgery, University Hospital of North Norway, Tromsø, Norway; § Department of Radiology, University Hospital of North Norway, Tromsø, Norway.

## Abstract

A bronchopleural fistula (BF) is a life-threatening complication. Optimal management of a BF is still debated although surgery remains the preferred treatment. Usually, the fistula is a result of inadequate healing at the bronchial stump after pneumonectomy. Successful closure of a BF after pneumonectomy depends on evacuation of empyema, coverage of the suture line after fistula closure with vascularized tissue, and obliteration of the residual pleural cavity. Extrathoracic muscles and omentum are the first choice for intrathoracal transposition. We report a unique case of a cachectic female patient with a BF from the left main stem bronchus complicated with empyema following right-sided pneumonectomy. Previous surgeries excluded the use of extrathoracic muscles or only omentum. The BF could not be closed with sutures. Using a parachute technique, omentum was sutured into the fistula opening resulting in a tension-free fistula closure. A well-vascularized breast was transposed into the residual pleural cavity to obliterate dead space and to support the omentoplasty, so it would be able to withstand changes in intrathoracic pressure. The postoperative course was uneventful. Tension-free closure of a BF can be obtained by suturing well-vascularized tissue into the fistula opening using a parachute technique. Intrathoracic breast transposition could be a new option in the treatment of a BF and associated empyema in a female patient. In selected patients, a large breast can obliterate the dead space after pneumonectomy and support the omentoplasty.

## INTRODUCTION

A bronchopleural fistula (BF) is a life-threatening complication.^1^ The optimal management of BF is still debated although surgery remains the preferred treatment. Usually, the fistula is a result of inadequate healing at the bronchial stump after pneumonectomy. We report a unique case of a BF from the left main stem bronchus complicated with empyema following a right-sided pneumonectomy for cancer surgery. Tissue damage from previous surgeries disallowed the use of extrathoracic muscles for traditional intrathoracic muscle transposition. Due to the patient’s malnourished state and previous abdominal surgery, omentoplasty alone was considered associated with a high risk for failure as it would not provide enough volume to obliterate dead space and would not be able to withstand changes of intrathoracic pressure when used for BF closure.

## CASE PRESENTATION

A 77-year-old woman was diagnosed in 2001 with a leiomyosarcoma of her left kidney primarily treated with a nephrectomy through a Chevron incision. In 2009, routine control uncovered 3 metastases in the left lung and 1 in the right lung, which were removed by segmentectomy through a median sternotomy. A thoracotomy was performed in 2013 for a pulmonary metastasis on the right side, and in 2016 for a metastasis on the left side.

In 2018, a right upper lobectomy was carried out through a thoracotomy to remove metastases. Postoperative air leakage from the lower lobes and bronchial stump required a reoperation. The leaks were closed with sutures, which were covered with a fibrin sealant patch (TachoSil, Nycomed Linz, Austria). Postoperatively, a chylothorax developed which was treated with parenteral nutrition. After 3 days, she developed septicemia. Bacteriological cultures of the pleural cavity revealed *Enterobacter cloacae*. Antibiotic treatment was adjusted, and her condition improved. However, air leakage recurred and increased during consecutive days. At reoperation, a right-sided pneumonectomy was performed. Intraoperatively, the left main stem bronchus was damaged due to manipulation while mobilizing the right main stem bronchus near the carina. The defect was closed with sutures. Postoperatively, a BF of the left main stem bronchus developed in combination with empyema. A reoperation was scheduled.

## SURGICAL PROCEDURE

**Fig. 1.**
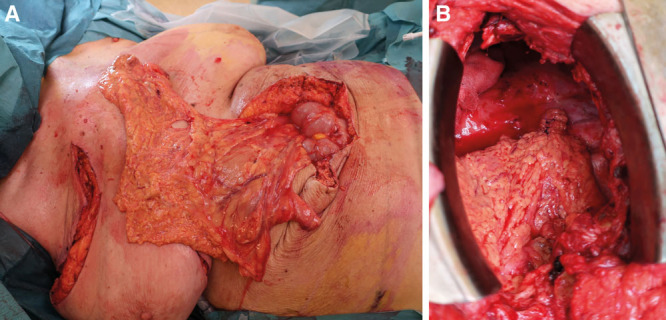
Omentum (A) was sutured into the BF opening using a parachute technique (B).

**Fig. 2.**
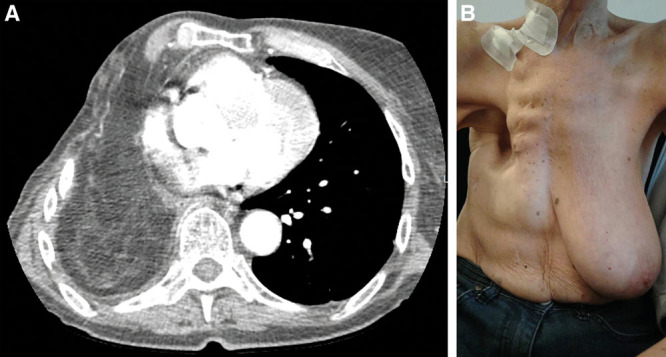
The computed tomography scan (A) shows the breast in the pleural cavity and placed against the omentum. B, The patient at 12-month follow-up.

In the supine position, a flimsy omentum was harvested through a laparotomy (Fig. 1). In the lateral decubitus position, the thoracotomy incision was reopened. Debridement was performed revealing a BF with a diameter of 8 mm. Tension-free BF closure with sutures was impossible. Omentum was tunneled into the pleural cavity by creating an opening in the diaphragm. Using a parachute technique, the omentum was sutured into the fistula opening as a plug obtaining a tension-free closure (Fig. 1). The right breast was completely harvested by creating a medial and lateral pedicle on its intercostal and internal mammary perforators, respectively, and leaving its blood supply from the underlying pectoralis major muscle also intact. The pectoralis major muscle was released from the sternum and ribs. The breast was deepithelialized. A segment of the fourth rib was removed. The whole breast, hinged on its pedicles and pectoralis major muscle, was sunken into the pleural cavity and placed upon the omentoplasty (Fig. 2). The thoracotomy wound was closed over a drain. The patient was extubated at the end of surgery. The postoperative course was uneventful (Fig. 2).

## DISCUSSION

Successful BF closure depends on control of infection and air leak, coverage of the suture line after BF closure with well-vascularized tissue, and obliteration of the residual pleural cavity.^1^ Early surgical intervention prevents deterioration of the patients’ condition. Jack mentioned 3 factors that may contribute to the breakdown of a suture line: an inadequate blood supply, tension on the sutures, and infection.^2^

No tension-free closure could be obtained in our patient by BF closure with sutures. Instead, we sutured omentum into the BF opening using a parachute technique. The use of this technique provided a tension-free BF closure.

Puskas et al found omentum to be superior to muscle flaps for coverage of the suture line after BF closure, reporting a 92% success rate for omentum versus 64% for muscle.^3^ Omentum is an ideal flap as it induces angiogenesis and functions in the presence of infection, and its pliability allows intimate contact with the fistula wall. Nevertheless, a failure rate of 8% has been reported for this flap.^3^

To obtain BF healing, omentum must adhere to the defect and be able to withstand changes in intrathoracic pressure. To reinforce the omentoplasty and to obliterate the residual pleural space, we transposed a well-vascularized breast into the pleural cavity and positioned it against the omentum. Strombeck showed the reliability of this bipedicled breast flap in mammaplasty.^4^ Although intrathoracic muscle transposition is the most effective method to obliterate the residual pleural space, the main blood supply to the various thoracic muscles and the rectus abdominis muscles in our patient were damaged by previous thoracotomies and the abdominal Chevron incision, respectively, although their innervations were still intact. Our patient had considerable respiratory constraints, and using these muscles would have aggravated her respiratory distress. Omentum provides only 5%–15% filling of the total pneumonectomy space.^4^ Due to malnourishment, omentum in our patient provided an even smaller volume.

Our patient was in a debilitated condition after the complicated postoperative course of the primary operation. Low albumin, age over 60 years, and impaired lung function made the risk for postoperative complications high.^5^ The BF at the main stem bronchus of the healthy left lung increased the risk for aspiration pneumonia.

We were aiming for a procedure that would reduce the risk for postoperative complications. Intrathoracic breast transposition in a female patient is easy to perform and can, in selected female patients, provide a large volume with well-vascularized tissue to support an omentoplasty and to fill the residual pleural cavity. To our knowledge, our procedure has not been reported before.

## ACKNOWLEDGMENT

We thank Dr. OA Østerud, MD, from the Department of Plastic Surgery, Mosjøen, Norway, for the postoperative photograph.

## References

[1] Bribriesco A Patterson GA Management of postpneumonectomy bronchopleural fistula: from thoracoplasty to transsternal closure. Thorac Surg Clin. 2018;28:323–335. 30054070 10.1016/j.thorsurg.2018.05.008

[2] Jack GD Bronchial closure. Thorax. 1965;20:8–12. 14255492 10.1136/thx.20.1.8 PMC1018887

[3] Puskas JD Mathisen DJ Grillo HC Treatment strategies for bronchopleural fistula. J Thorac Cardiovasc Surg. 1995;109:989–995; discussion 995. 7739261 10.1016/S0022-5223(95)70325-X

[4] Strombeck JO Mammaplasty: report of a new technique based on the two-pedicle procedure. Br J Plast Surg. 1960;13:79–90. 13835285 10.1016/s0007-1226(60)80014-8

[5] Miller JI Mansour KA Nahai F Single-stage complete muscle flap closure of the postpneumonectomy empyema space: a new method and possible solution to a disturbing complication. Ann Thorac Surg. 1984;38:227–231. 6236761 10.1016/s0003-4975(10)62243-6

